# Effects of an Advanced Clinical Practice Nurse-Led Discharge Management and Education Program on Patient Outcomes in an Acute Medical Care Unit (ADIEU)

**DOI:** 10.1155/jonm/5425868

**Published:** 2025-11-16

**Authors:** Seungjin Lee, Ji Eun Song, Seunghyun Won, Nak-Hyun Kim, Jung Hun Ohn, Yejee Lim, Jongchan Lee, Hye Won Kim, Sun-wook Kim, Jiwon Ryu, Hee-Sun Park, Jihye Kim, Yunsang Choi, Eun Sun Kim

**Affiliations:** ^1^Hospital Medicine Center, Seoul National University Bundang Hospital, Seongnam, Republic of Korea; ^2^College of Nursing, Seoul National University, Seoul, Republic of Korea; ^3^Division of Statistics, Medical Research Collaborating Center, Seoul National University Bundang Hospital, Seongnam, Republic of Korea; ^4^Department of Internal Medicine, Seoul National University Bundang Hospital, Seoul National University College of Medicine, Seongnam, Republic of Korea

## Abstract

**Background:**

Patients in acute medical care units (AMUs) often encounter complex discharge processes due to rapid turnover and acute care demands. The advanced clinical practice nurse-led discharge management and education (ADIEU) program was developed to address these challenges and improve postdischarge outcomes.

**Aims:**

To evaluate the effectiveness of the ADIEU program on clinical outcomes and satisfaction among patients and healthcare providers in AMUs.

**Methods:**

This retrospective study enrolled 360 patients discharged from the AMU of a tertiary hospital in Republic of Korea and compared them before and after ADIEU program implementation. Propensity score matching (PSM) was applied using age, sex, Charlson Comorbidity Index (CCI), and reason for admission as matching variables. After PSM, 139 patients were included in each group. Thirty-day readmissions and unexpected emergency department (ED) visits within 72 h were evaluated after PSM. Patient and healthcare personnel satisfaction were separately assessed.

**Results:**

The ADIEU program significantly reduced the risk of 30-day readmission (odds ratio = 0.333). However, no significant reduction in unexpected ED visits within 72 h was observed. Discharge management satisfaction improved among nurses (mean score: 3.09–3.43) and physicians (mean score: 3.67–4.67).

**Conclusions:**

The ADIEU program effectively reduced 30-day readmissions and increased satisfaction among healthcare providers and patients.

**Implications for Nursing Management:**

The ADIEU program represents a promising model for enhancing discharge management in AMUs despite its minimal impact on short-term ED visits.

## 1. Introduction

Patient discharge is a crucial aspect of healthcare management, as it is vital for allocating bed spaces and maintaining the efficient functioning of multidisciplinary treatment teams. The discharge process requires coordinated efforts among medical and nursing staff to ensure continuity and safety of care [1] and involves the meticulous transition of patients from hospitals to community settings as their condition improves. Effective discharge planning includes a multidisciplinary approach, assessment integration, goal setting, planning, implementation, and evaluation to ensure continuity of care [2]. Furthermore, it necessitates comprehensive communication among the medical staff responsible for the patient's care.

Previous studies have shown that patients often experience the highest incidence of complications, medication errors, and unplanned healthcare utilization within the first month after discharge, underscoring the critical need for structured discharge planning and education during this period [3, 4]. In addition, approximately 20% of hospitalizations are associated with adverse events postdischarge, and some can result in preventable readmissions or emergency department (ED) visits [1, 5]. This highlights the significant burden of postdischarge care on patients and their families as they reintegrate into the community with complex health conditions [6]. Recently, a prospective cohort study described the problems with patients' lack of understanding regarding postdischarge care [7]. Therefore, individualized counseling and education programs are crucial to support self-management after discharge and aim to reduce readmission rates and avoidable ED visits. In support of these efforts, several recent studies have reported that implementing various discharge management programs in general hospital wards has effectively reduced patients' ED visits and readmission rates [1, 8]. However, studies investigating discharge management programs focused on patients discharged from acute medical care units (AMUs) are limited.

AMUs, implemented globally, including in Korea, are designed to efficiently evaluate and provide comprehensive care for patients with acute medical illnesses, thereby optimizing bed management and enhancing patient flow [9]. These units prioritize prompt treatment from admission, aiming for hospital stays < 72 h, with 30%–60% discharged directly, while particularly benefiting patients with severe chronic or acute conditions [9–11]. AMUs are praised for their efficiency in the treatment and management of various medical diseases with a focus on short-term hospitalization; however, they present with distinct challenges in safely transitioning patients from the hospital to home or existing outpatient clinics for follow-up, compared with traditional general wards [12].

Seoul National University Bundang Hospital (SNUBH) in South Korea established an AMU in 2015, where advanced clinical practice nurses (CPNs) and hospitalists collaborate for patient care. The hospital launched the advanced clinical practice nurse-led discharge management and education (ADIEU) program in January 2023, after acknowledging the challenges and significance of managing discharged patients. The discharge management and education were coordinated by CPNs collaborating with the hospitalist and used a discharge management form integrated into the electronic medical record (EMR) system. This approach was aimed at enhancing communication among the nurse, patient, and guardian.

In this study, we aimed to evaluate the impact of the ADIEU program on clinical outcomes among patients discharged following inpatient treatment at an AMU.

## 2. Methods

### 2.1. Study Design and Patient Population

We used a retrospective, before-and-after design to evaluate the impact of the ADIEU program on patient outcomes in the 46-bed AMU at SNUBH. The AMU staff is composed of a team of 10 internists, each with over 10 years of experience, and two advanced CPNs. It offers continuous care to adult patients with acute medical illnesses requiring hospitalization, most of whom are admitted through the ED. The ADIEU group consisted of patients directly discharged home from the AMU from January 1 to December 31, 2023. The control group included patients discharged between January 1 and December 31, 2022, prior to the program's implementation, to evaluate the effectiveness of the ADIEU program. Patients who required management and education of the six categories included in the ADIEU program were retrospectively reviewed.

### 2.2. Discharge Management and Education: The ADIEU Program

Beginning in January 2023, two advanced CPNs were assigned to the AMU to implement the ADIEU program. The CPNs participated in daily rounds with physicians, during which they confirmed the discharge plans. CPNs identified patients requiring discharge management and education in several areas, including catheter management, nutritional care, self-injection, oxygen therapy, wound care, and medication management. Patients discharged home with catheters, such as chest and intraperitoneal drainage tubes, were given individualized pamphlets and practiced dressing methods for catheter management. Nutritional care included guidance on increasing intake, maintaining appropriate diets, and training and precautions for patients requiring percutaneous endoscopic gastrostomy or percutaneous radiologic gastrostomy management. Training on methods of self-injection and precautionary education was given to patients requiring self-injection. For instance, patients prescribed subcutaneous injections such as insulin were trained in proper injection techniques and educated on precautions, including site rotation, recognition of local reactions, and safe disposal of sharps. Pamphlets on oxygen therapy were provided, and assistance with selecting and preparing oxygen equipment was offered, including verifying oxygen capacity and documentation. Education on wound care was provided to each patient, including basic information and caution on wound dressing. Medication management involved additional training for patients with difficulties in administering medications, dosage, and tapering; this training was conducted in collaboration with dedicated pharmacists.

The ADIEU program provided individualized education and management to patients 1–3 days before discharge. A discharge management form documenting the specific instructions and education provided was completed during these sessions. Subsequently, it was uploaded to the EMR to ensure effective communication within the multidisciplinary team. The discharge management form (Supporting Figure 1) includes information on family and social support structures, finances, daily and social activities, and the patient's readiness for, and understanding of, the discharge process. A patient satisfaction survey (Supporting Figure 2) was conducted on the day of discharge, either via short message service or in written form. In addition, telephone consultations were held when follow-up was required postdischarge.

### 2.3. Clinical Outcomes

Demographic information on sex, age, and underlying diseases and discharge records and prescription-related data at discharge were collected from each patient. Additional data were collected from discharge management forms and patient satisfaction surveys for patients who received ADIEU management. The primary outcome was the rate of ED revisits or unexpected outpatient department (OPD) visits within 72 h after discharge. Secondary outcomes included the readmission rate, number of unexpected ED or OPD visits within 30 days, and satisfaction levels of both patients and healthcare providers.

### 2.4. Statistical Analyses

Baseline characteristics were summarized as counts and percentages for categorical variables and as median [interquartile range] for continuous variables. Propensity score matching (PSM) was used to balance the groups (before and after the ADIEU program) based on matching variables, including sex, age, Charlson Comorbidity Index (CCI), and reasons for admission. The nearest-neighbor method with a 0.2 caliper width was used for PSM. Subsequently, univariable and multivariable logistic regression analyses were conducted to investigate the association between the ADIEU program and clinical outcomes. Variable selection was performed, following the inclusion of all baseline characteristics in the models. All statistical analyses were performed using the R Version 4.3.1 (the R Foundation for Statistical Computing, Vienna, Austria), with the MatchIt package for PSM [13]. Statistical significance was set at *p* < 0.05 (two-sided).

### 2.5. Ethical Considerations

The study protocol was approved by the institutional review board of our hospital. Informed consent was waived owing to the retrospective nature of the study and the use of deidentified patient data.

### 2.6. Reporting Method

The reporting followed the SQUIRE guideline.

## 3. Results

### 3.1. Patient Characteristics

**Figure 1:**
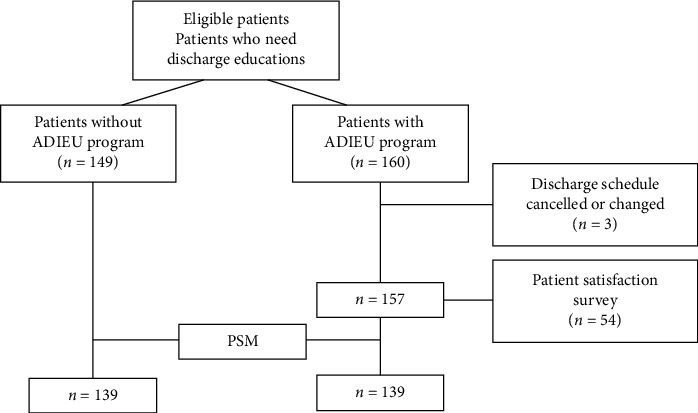
Patient enrollment process.

We enrolled 160 patients in the ADIEU program between January 1 and December 31, 2023, who required a discharge management and were discharged directly from the AMU. Three patients were excluded from the study because of transferred to other services or hospitals or discharge cancellation. Of the 157 eligible patients, 54 completed the satisfaction survey for the ADIEU program (Figure 1). Patient baseline characteristics, both before and after PSM, are summarized in Table 1. More than half of the patients were male (57.6%), with a median age of 71 years. Infectious diseases (35.3%) were the most common reason for admission to the AMU. Baseline variables between the two groups were evenly balanced after PSM (Table 1, Supporting Figure 3).

### 3.2. Discharge Management Form

Analysis of the discharge management forms revealed that two patients (1.3%) were living with neighbors or housemates. In contrast, 22 patients (14%) were living alone. The remaining patients (84.7%) lived with, and received support from, family members. At admission, 77.7% were cared for by their family. In addition, we confirmed that most patients (84.7%) were capable of performing activities of daily living independently without assistance.

The assessment of both patients' and caregivers' awareness regarding the disease status and discharge readiness is summarized in Table 2.

At the time of discharge, 47.8% of patients and 43.3% of caregivers had a clear understanding of the disease status; however, 14% of patients and 1.3% of caregivers reported no understanding. Furthermore, when patients were asked about their subjective readiness for discharge, 78.3% believed they could be immediately discharged, and 19.7% believed they could be discharged within 2-3 days. This indicated that 98% of patients believed they could be discharged at the point of management.

### 3.3. Discharge Education and Satisfaction Survey

**Figure 2:**
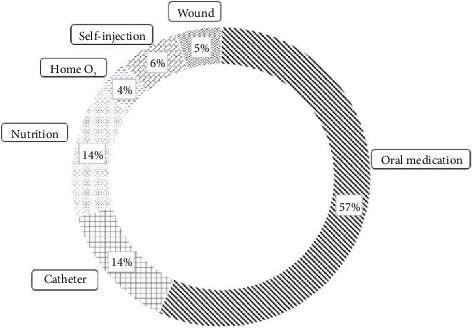
Distribution of discharge management and education in the advanced clinical practice nurse-led discharge management and education program.

Figure 2 shows the distribution of the six discharge management items received by patients who participated in the ADIEU program. The most common items were medication adjustment and administration and education on side effects (56.9%), followed by instructions on catheter management (13.8%) and nutritional management and support (14.2%).

The levels of satisfaction of healthcare providers and participants regarding the ADIEU program were measured using a Likert scale calibrated from 0 to 5, with 5 representing the highest level of satisfaction. Nurses (*n* = 23) reported an increase in satisfaction with discharge management, with their mean score increasing from 3.09 ± 0.73 preintervention to 3.43 ± 0.73 postintervention. Similarly, doctors (*n* = 9) showed notable improvement in the satisfaction score, which increased from 3.67 ± 1.22 preintervention to 4.67 ± 0.50 postintervention. Of the 157 participants in the ADIEU program, 54 completed the postprogram survey, yielding an average satisfaction score of 4.81 ± 0.52.

### 3.4. Clinical Outcomes

Supporting Table 1 summarizes the univariable logistic regression analysis for the unexpected ED visit outcomes within 72 h, readmission within 30 days, and a composite outcome of these two variables after PSM. The ADIEU program was significantly associated with a reduction in readmission within 30 days (odds ratio [OR] = 0.357, 95% confidence interval [CI]: 0.169–0.751, *p*=0.007). Furthermore, the composite outcome of unexpected ED visits within 72 h or readmission significantly reduced with the program (OR = 0.292, 95% CI: 0.144–0.592, *p* < 0.001). However, unexpected ED visits within 72 h alone were not significantly different between the two groups.

Table 3 presents the results of the multivariable logistic regression analysis, adjusted for age, sex, BMI, CCI, and reason for admission. After variable adjustment, the ADIEU program showed a statistically significant reduction in readmission within 30 days (OR = 0.333, 95% CI: 0.154–0.720, *p*=0.005; Table 3). Furthermore, the program significantly reduced the composite outcome of unexpected ED visits within 72 h or readmission (OR = 0.275, 95% CI: 0.133–0.574, *p*=0.001; Table 3). CCI was identified as a significant predictor for both outcomes among the adjusted variables. Specifically, a higher CCI was associated with an increased risk of readmission (OR = 1.241, 95% CI: 1.076–1.432, *p*=0.003) and the composite outcome (OR = 1.201, 95% CI: 1.054–1.367, *p*=0.006).

## 4. Discussion

This study represents a pioneering effort in implementing the ADIEU discharge management program, tailored for patients admitted to an AMU in Korea. The ADIEU program demonstrated significant efficacy in reducing readmissions within 30 days and achieved high satisfaction ratings from both healthcare workers and patients, consistent with previous studies' findings conducted in general wards [14–16]. AMUs efficiently reduce the length of stay and in-hospital mortality [12]. However, they present unique challenges to patient safety, particularly in ensuring smooth and secure transition from hospital to home or outpatient care. These challenges are heightened by the rapid patient turnover and acute care focus that characterizes AMUs, which can increase the risk of postdischarge complications if not properly managed. Our findings indicate that the ADIEU program enhances patient safety by addressing gaps in postdischarge care, leading to safer transitions and improved outcomes. This emphasizes the importance of incorporating discharge management programs in AMUs to support patients during the critical transition from hospital to home.

In this study, the rate of unexpected ED visits within 72 h did not differ between the groups that participated in the ADIEU program and those who did not, compared with the significant reduction in unplanned readmission within 30 days. One possible explanation is that 72 h could be insufficient for the manifestation of postdischarge complications. Notably, before the implementation of the ADIEU program, patients in the AMU received comprehensive discharge instructions from nursing staff, attending physicians, and dedicated pharmacists, which may have mitigated the risk of immediate postdischarge complications. Consequently, complications requiring an ED visit may not arise within such a short period. Notably, a randomized controlled trial that implemented discharge education in the ED—a setting characterized by similarly rapid patient turnover and acute care focus—used a longer follow-up window of 30 or 90 days for measuring unexpected ED revisits [11, 17]. Although the rate of unexpected ED visits within 72 h was not significantly different between the groups, this finding provides important insight into the potential limitations of short-term interventions and highlights the need for continuous follow-up strategies.

Notably, although 98% of patients indicated readiness for discharge either immediately or within 2-3 days, less than half of them demonstrated a clear understanding of their current diseases and medical instructions after discharge. The findings on discharge readiness and management revealed significant gaps in the patients' actual preparedness despite the fact that 78.3% of patients felt ready for discharge and rated their subjective condition highly. This observation is consistent with a previous research that reported that only approximately half of patients fully understood their reason for admission or medical history [7]. This gap indicates that patients often equate feeling well with being ready for discharge, potentially overlooking the need for ongoing care. Therefore, ensuring that both patients and caregivers have a comprehensive understanding of the patient's condition through precise communication, repeated education, and thorough assessments of discharge readiness is crucial.

This study is notable for its innovative approach to evaluating the ADIEU program specifically for patients in an AMU. It addresses a critical gap in research by focusing on structured discharge interventions for patients in an AMU. The ADIEU program uses a comprehensive methodology, compared with traditional discharge education methods [14], which usually involve fragmented approaches such as providing verbal instructions at discharge, issuing discharge letters, or making follow-up telephone calls. It systematically assesses patients' socioeconomic conditions, evaluates their understanding of their medical condition and discharge readiness, and provides targeted education in six areas commonly associated with postdischarge complications. The structured and comprehensive approach of the ADIEU program represents a significant advancement over existing practices and demonstrates its potential to enhance patient outcome and safety. In addition, the program was implemented through collaboration between CPNs and hospitalists, emphasizing the potential benefits of teamwork in improving the quality of patient care.

Despite the strengths of this study, this study has some limitations. The evaluation of clinical outcomes associated with the ADIEU program was conducted retrospectively using PSM. To better assess the program's clinical impact, a study design with greater statistical rigor, such as a prospective randomized controlled trial, would have been preferred. However, given that the ADIEU program was part of a quality improvement initiative and was analyzed retrospectively, implementing such a design was not feasible. Therefore, future studies should endeavor to address this gap with a well-designed prospective study. This study was performed in a single AMU within a quaternary hospital in South Korea, involving a small patient sample. Consequently, the findings may not be generalizable to other settings or healthcare systems. To enhance the generalizability of the results, conducting a multicenter study with diverse populations and settings would be beneficial. Lastly, although the ADIEU program addressed six key areas of discharge management, it is possible that some important aspects were not covered. In addition, there were no follow-up measures, such as postdischarge evaluations or telephone calls, to verify if patients and caregivers properly understood the education provided. To address these gaps, we intend to continue quality improvement activities and enhance the program to cover any identified deficiencies. Conducting a follow-up study to evaluate these improvements would be beneficial in assessing the program's overall effectiveness and ensuring better postdischarge outcomes.

## 5. Conclusions

The ADIEU program effectively enhances patient outcomes and satisfaction by encouraging teamwork, improving communication, and adopting a comprehensive, patient-centered approach to discharge planning. Despite its significant benefits, the program has some limitations; therefore, further research is important to assess its applicability in diverse healthcare settings. Continued efforts to refine and adapt the ADIEU program will be essential in maximizing its effectiveness and ensuring optimal patient care.

## Figures and Tables

**Table 1:** Baseline characteristics of patients before and after propensity score matching.

	Before matching		After matching		*p* value
	Before ADIEU program (*n* = 149)	After ADIEU program (*n* = 157)	Before ADIEU program (*n* = 139)	After ADIEU program (*n* = 139)	
Sex (male)	81 (54.4)	89 (56.7)	76 (54.7)	80 (57.6)	0.717
Age (years)	71.00 [62.00–78.00]	71.00 [65.00–81.00]	71.00 [63.00–79.00]	71.00 [65.00–81.00]	0.561
Body weight (kg)	57.00 [49.80–64.00]	60.00 [51.00–67.20]	58.20 [50.75–64.45]	60.00 [52.75–66.75]	0.112
BMI (kg/m^2^)	22.10 [19.90–25.20]	22.70 [20.80–25.90]	22.10 [20.00–25.20]	22.70 [20.90–25.90]	0.206
CCI	7.00 [4.00–9.00]	7.00 [4.00–9.00]	7.00 [4.00–9.00]	7.00 [4.00–9.00]	0.896
Reason for admission					0.987
Infection	47 (31.5)	59 (37.6)	47 (33.8)	49 (35.3)	
Workup	26 (17.4)	28 (17.8)	26 (18.7)	24 (17.3)	
Supportive care	20 (13.4)	20 (12.7)	18 (12.9)	19 (13.7)	
Noninfectious complication of previous disease	56 (37.6)	50 (31.8)	48 (34.5)	47 (33.8)	

*Note:* Values are presented as *n* (%) or median [interquartile range].

Abbreviations: BMI = body mass index; CCI = Charlson Comorbidity Index.

**Table 2:** Awareness regarding the disease status and discharge readiness in the discharge management form (*n* = 157).

Questions	
Patient's understanding of their disease	
Fully understands	75 (47.8)
Partially understands	47 (29.9)
Cannot understand	22 (14.0)
N/A	13 (8.3)
Caregiver's understanding of the patient's disease	
Fully understands	68 (43.3)
Partially understands	38 (24.2)
Cannot understand	2 (1.3)
N/A	49 (31.2)
Patient's subject readiness for discharge	
Ready to discharge tomorrow	123 (78.3)
Ready in 2-3 days	31 (19.7)
Ready after 1 week	3 (1.9)
Caregiver after discharge (multiple selection allowed)	
Family	111 (70.7)
Friend	2 (1.3)
UAP	9 (5.7)
Caregiver	4 (2.5)
None	31 (19.7)
Need medical support after discharge	
N/A	133 (84.7)
Home nursing	16 (10.2)
Local hospital	8 (5.1)
Patient's subject condition for discharge	9.00 [6.00–10.00]
Awareness for medical caution	
Very well	32 (20.4)
Somewhat	67 (42.7)
Do not know well	46 (29.3)
Do not know at all	11 (7.0)
N/A	1 (0.6)
Subject readiness for ADL	
Very well	69 (43.9)
Somewhat (can do > 50%)	64 (40.8)
Cannot do well (< 50%)	10 (6.4)
Cannot do at all	14 (8.9)

*Note:* Values are presented as *n* (%) or median [interquartile range].

Abbreviations: ADL = activities of daily living, N/A = not applicable (cases in which the patient cannot communicate or the caregiver was absent), and UAP = unlicensed assistive personnel.

**Table 3:** Multivariable logistic regression analysis in the ADIEU program after propensity score matching.

	OR	95% CI	*p* value
Unexpected ED visit within 72 h			
Post-ADIEU group (Ref: pre-ADIEU)	0.576	0.182–1.828	0.349
Age	1.006	0.954–1.061	0.817
Sex (male)	0.496	0.145–1.704	0.266
BMI	0.857	0.724–1.014	0.073
CCI	1.097	0.897–1.343	0.368
Reason for admission (Ref: infectious problem)			
Workup	0.771	0.185–3.215	0.721
Supportive care	0.212	0.023–1.938	0.170
Noninfectious complication of previous disease	0.295	0.070–1.240	0.096
Readmission in 30 days			
Post-ADIEU group (Ref: pre-ADIEU)	0.333	0.154–0.720	0.005
Age	0.996	0.962–1.032	0.842
Sex (male)	0.839	0.400–1.763	0.644
BMI	0.970	0.883–1.066	0.529
CCI	1.241	1.076–1.432	0.003
Reason for admission (Ref: infectious problem)			
Workup	1.176	0.407–3.396	0.765
Supportive care	1.824	0.624–5.331	0.272
Noninfectious complication of previous disease	0.932	0.375–2.319	0.881
Composite outcome (unexpected ED visit within 72 h or readmission in 30 days)			
Post-ADIEU group (Ref: pre-ADIEU)	0.275	0.133–0.574	0.001
Age	0.998	0.966–1.030	0.887
Sex (male)	0.795	0.398–1.590	0.517
BMI	0.950	0.869–1.038	0.254
CCI	1.201	1.054–1.367	0.006
Reason for admission (Ref: infectious problem)			
Workup	0.885	0.334–2.346	0.806
Supportive care	1.296	0.476–3.526	0.611
Noninfectious complication of previous disease	0.634	0.274–1.470	0.289

Abbreviations: 95% CI = confidence interval, ADIEU = advanced clinical practice nurse-led discharge management and education program, BMI = body mass index, CCI = Charlson Comorbidity Index, ED = emergency department, OR = odds ratio, and Ref = reference.

## Data Availability

The datasets generated and/or analyzed during the current study are not publicly available due to restrictions on data outflow from the hospital but are available from the corresponding author on reasonable request.

## Supporting Information

Supporting Table 1: univariable logistic regression analysis in the ADIEU program after propensity score matching.

Supporting Figure 1: discharge management counseling record.

Supporting Figure 2: discharge counseling and education survey.

Supporting Figure 3: balancing plot of the patients before and after propensity score matching.
