# Supplementary material for: Effects of an Advanced Clinical Practice Nurse-Led Discharge Management and Education Program on Patient Outcomes in an Acute Medical Care Unit (ADIEU)
Source: J Nurs Manag. 2025 Nov 16;2025:5425868. doi: 10.1155/jonm/5425868 (PMC12640755; doi:10.1155/jonm/5425868)
Supplement: Supporting Information — Additional supporting information can be found online in the Supporting Information section. [file 5425868.f1.docx]

**Supplementary Table 1. Univariable Logistic Regression Analysis in the ADEIU Program after Propensity Score Matching**

a) Unexpected ER visit within 72hrs

|  | OR | 95% CI | p-value |
| --- | --- | --- | --- |
|  | Unexpected ER visit within 72hrs | | |
| Post-ADEIU group  (Ref: Pre-ADEIU) | 0.539 | 0.176-1.651 | 0.279 |
| Age, years | 1.007 | 0.964 -0.964 | 0.759 |
| Gender, male | 0.495 | 0.151-1.618 | 0.245 |
| BMI | 0.883 | 0.767-1.018 | 0.087 |
| CCI | 1.102 | 0.915-1.328 | 0.305 |
| Admission reason  (Ref: Infectious problem) |  |  |  |
| Work up | 0.811 | 0.201-3.284 | 0.770 |
| Supportive care | 0.354 | 0.042-2.974 | 0.338 |
| Noninfectious complication of prev. disease | 0.415 | 0.104-1.654 | 0.212 |

**b) Readmission within 30days**

|  | OR | 95% CI | p-value |
| --- | --- | --- | --- |
|  | Readmission in 30 days | | |
| Post-ADEIU group  (Ref: Pre-ADEIU) | 0.357 | 0.169-0.751 | 0.007 |
| Age, years | 1.012 | 0.984-1.041 | 0.390 |
| Gender, male | 0.811 | 0.403-1.630 | 0.556 |
| BMI | 0.943 | 0.865-1.029 | 0.189 |
| CCI | 1.223 | 1.076-1.389 | 0.002 |
| Admission reason  (Ref: Infectious problem) |  |  |  |
| Work up | 1.257 | 0.455-3.475 | 0.658 |
| Supportive care | 2.132 | 0.781-5.815 | 0.139 |
| Noninfectious complication of prev. disease | 1.117 | 0.467-2.673 | 0.803 |

**c) Composite outcome regarding unexpected ER visit within 72hrs or readmission in 30 days**

|  | OR | 95% CI | p-value |
| --- | --- | --- | --- |
|  | Composite outcome  (Unexpected ER visit within 72hrsor Readmission in 30 days) | | |
| Post-ADEIU group (Ref:Pre-ADEIU) | 0.292 | 0.144-0.592 | <.001 |
| Age, years | 1.010 | 0.984-1.036 | 0.461 |
| Gender, male | 0.791 | 0.415-1.510 | 0.477 |
| BMI | 0.929 | 0.856-1.008 | 0.076 |
| CCI | 1.179 | 1.051-1.322 | 0.005 |
| Admission reason  (Ref: Infectious problem) |  |  |  |
| Work up | 0.952 | 0.377-2.407 | 0.918 |
| Supportive care | 1.606 | 0.639-4.045 | 0.314 |
| Noninfectious complication of prev. disease | 0.793 | 0.358-1.754 | 0.566 |

95% CI, confidence interval

BMI, body mass index; CCI, Charlson Comorbidity Index

**Supplementary Figure 1. Discharge Management Counseling Record**

**
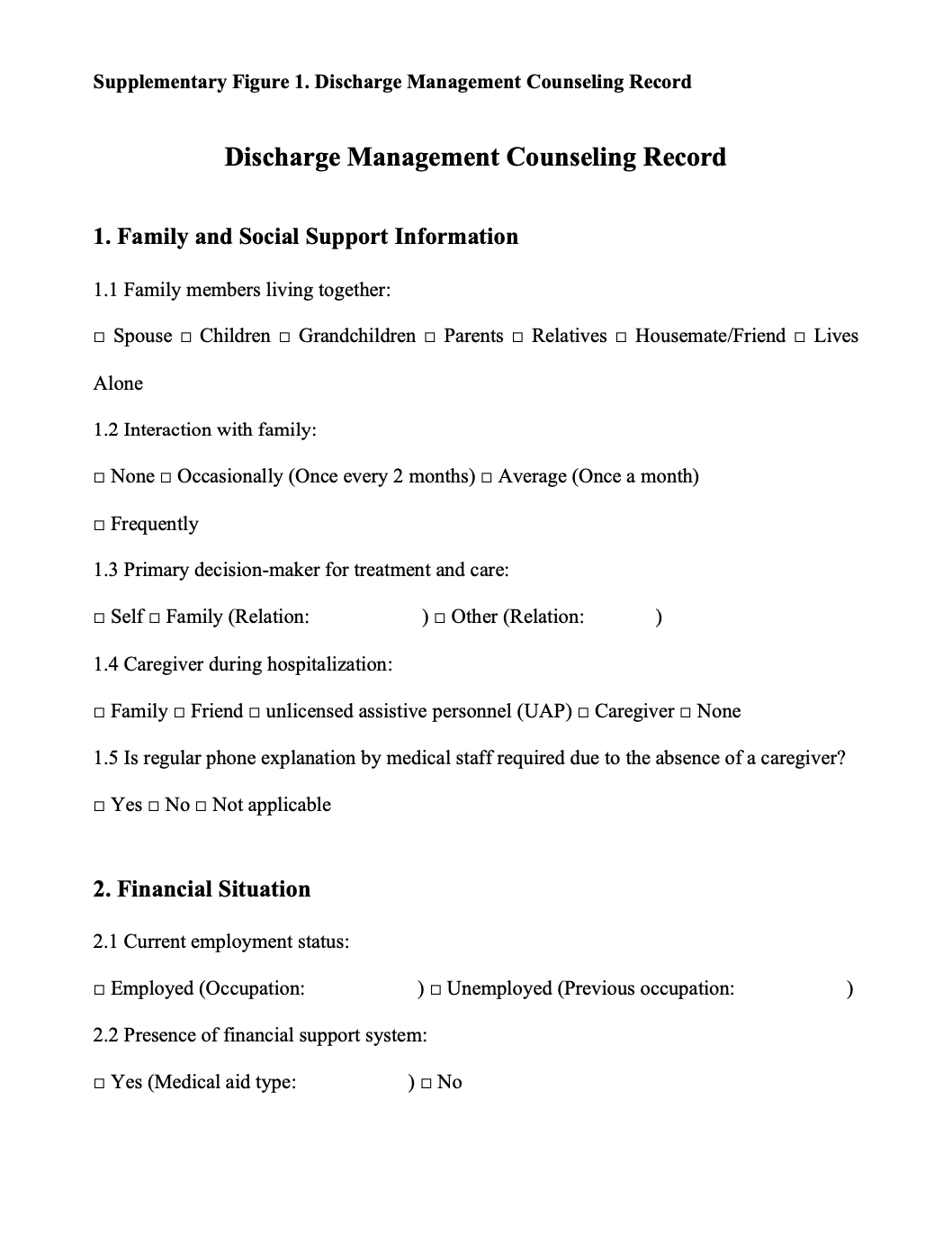
**

**
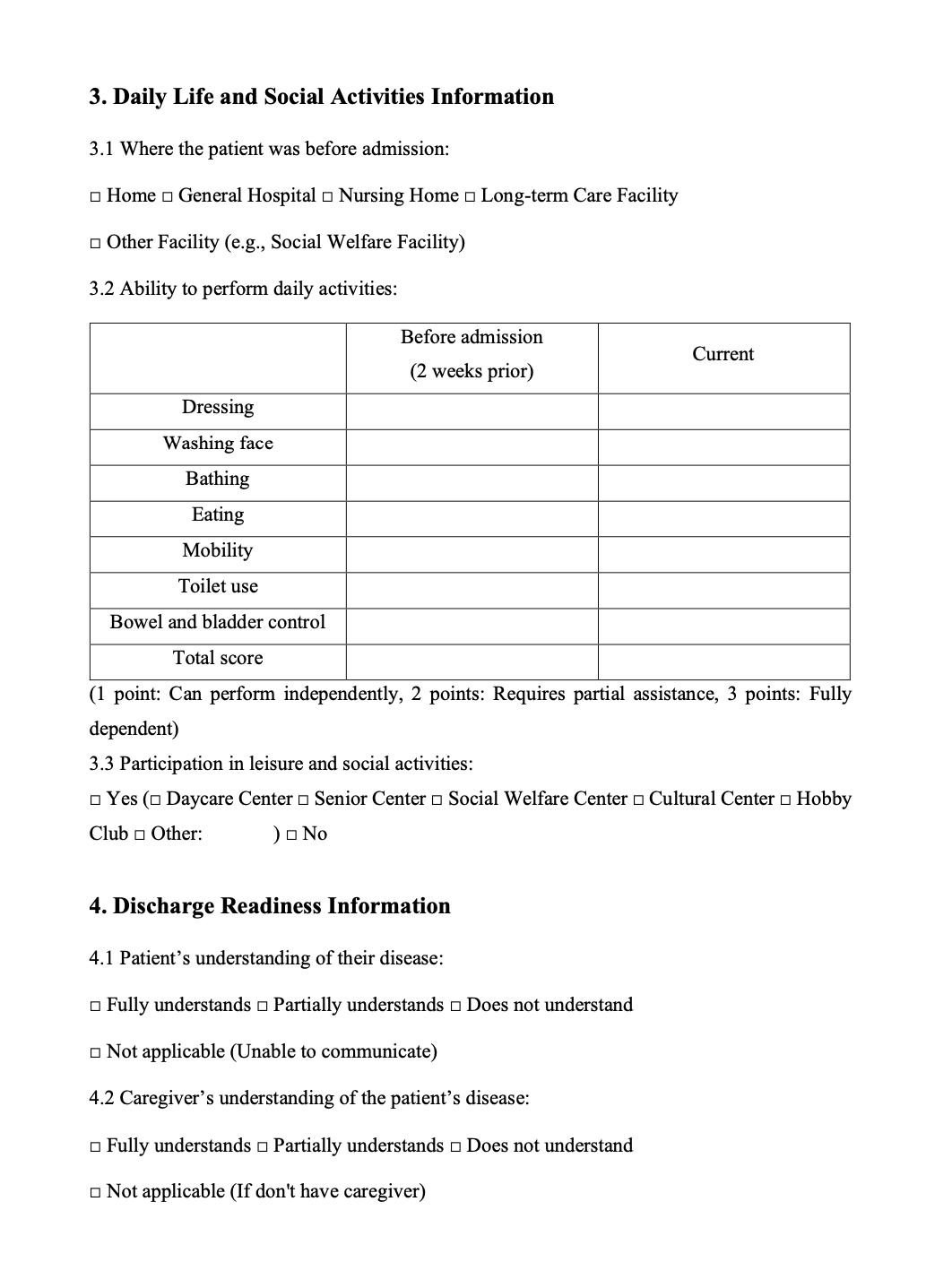
**

**
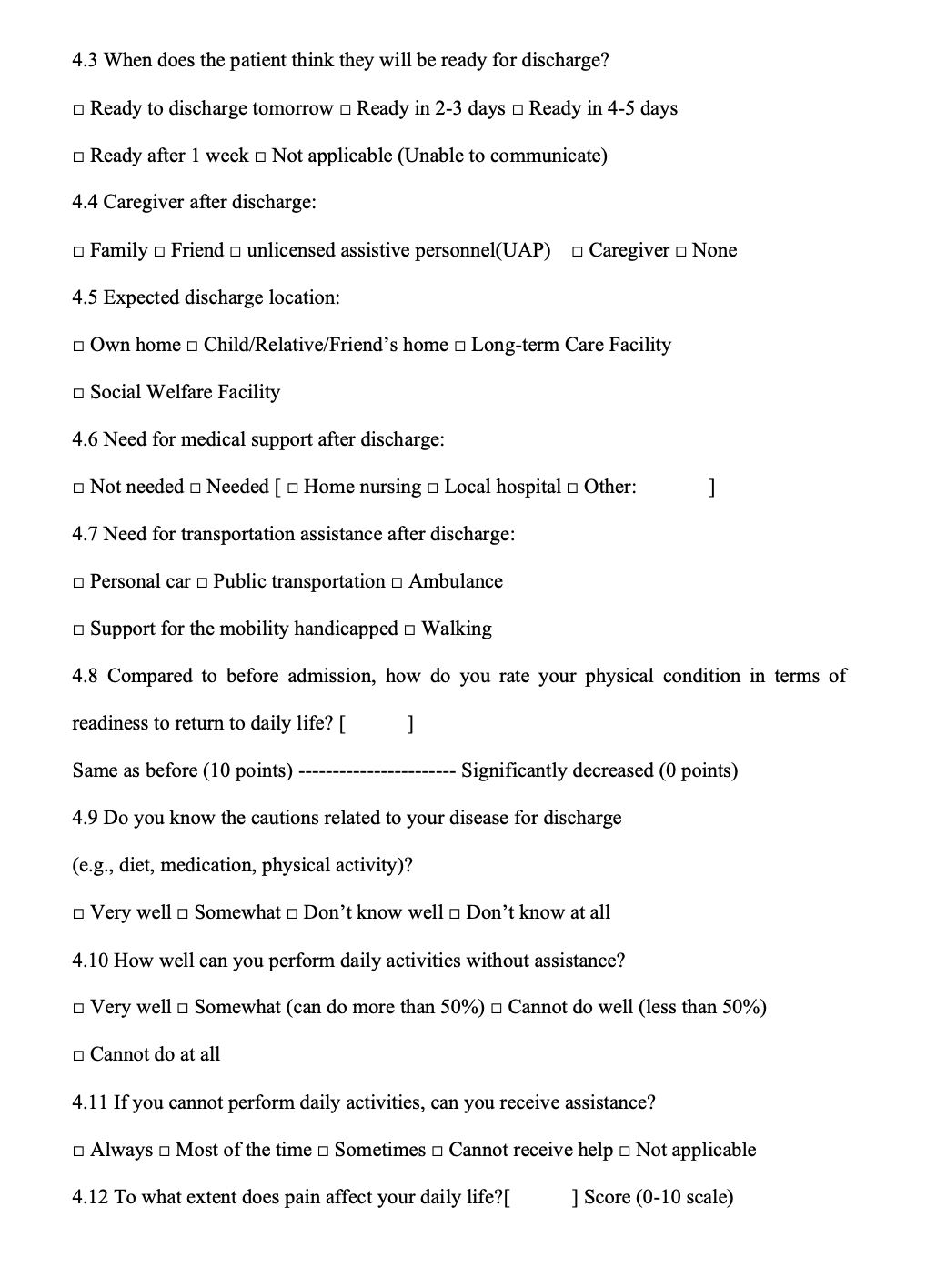

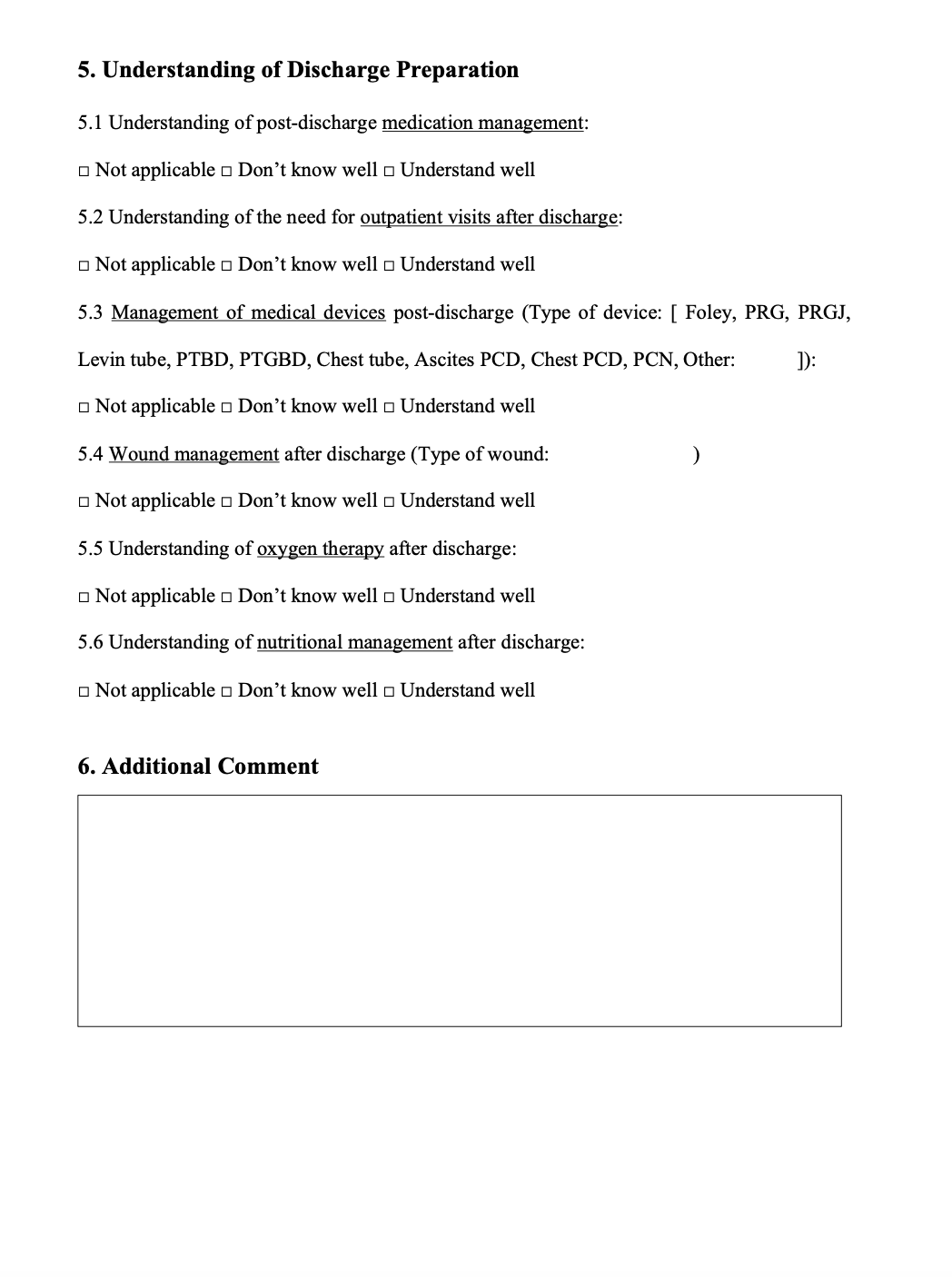
 Supplementary Figure 2. Discharge Counseling and Education Survey**


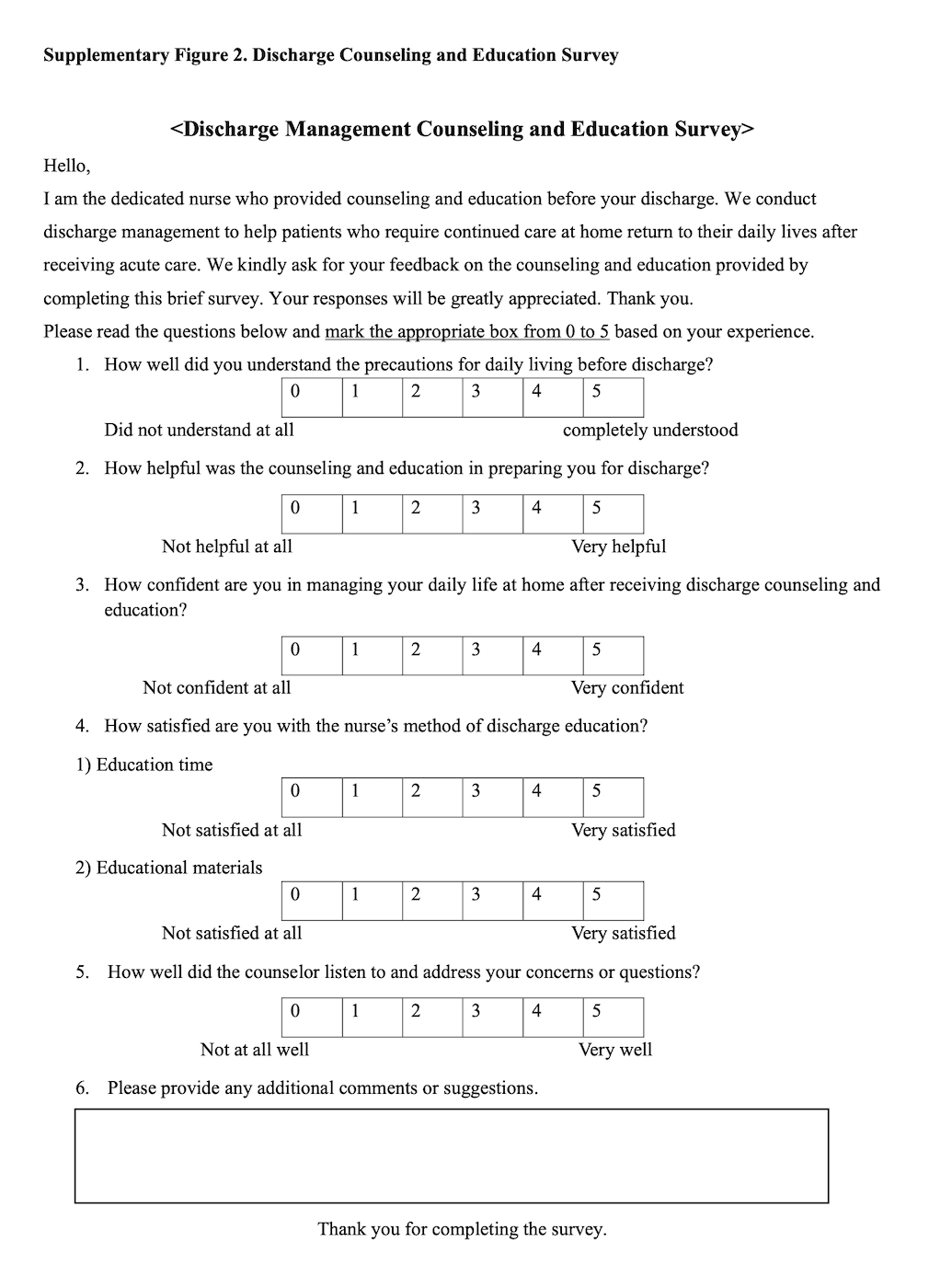
**Supplementary Figure 3. Balancing plot of the patients before and after propensity score matching**


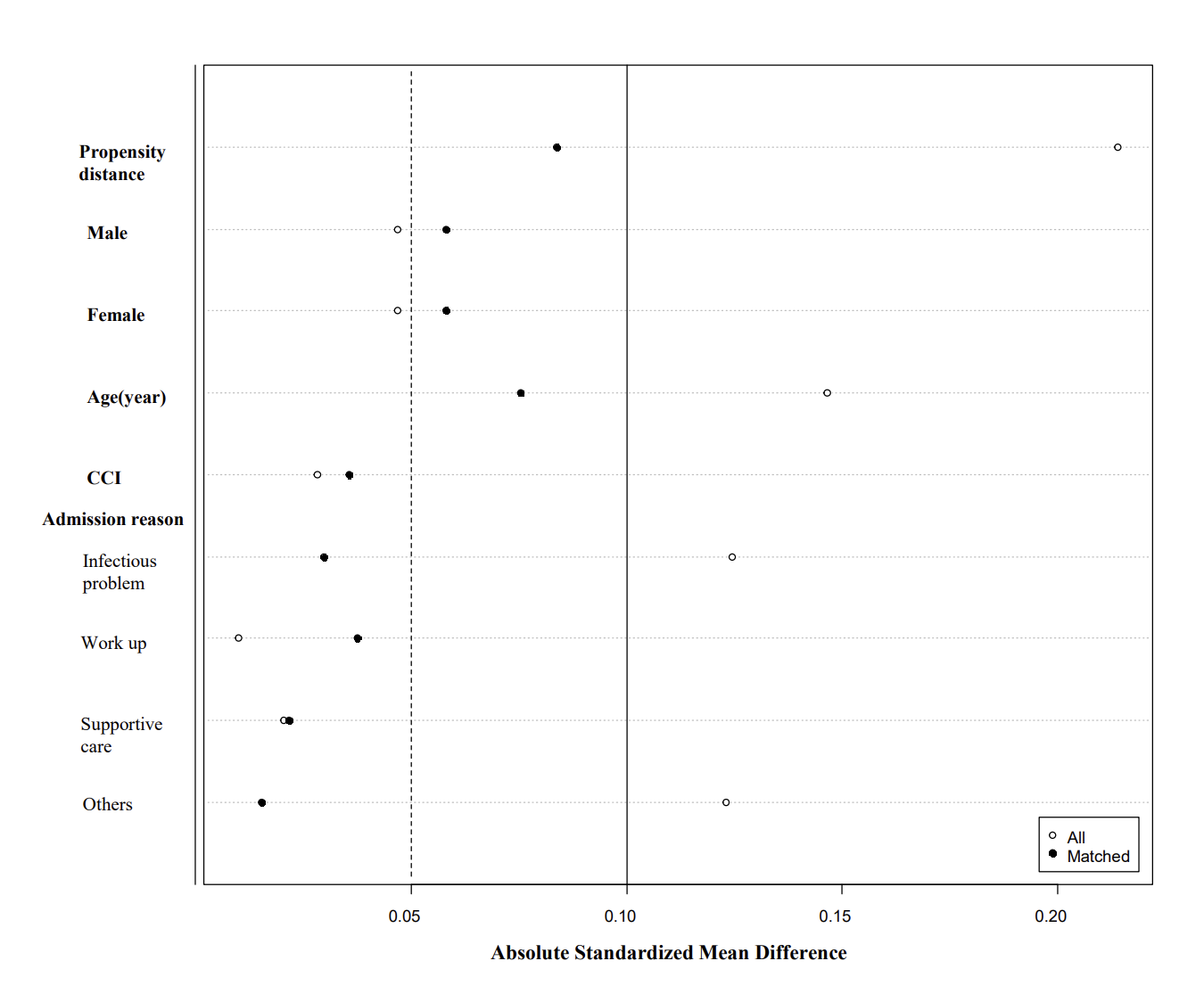
CCI, Charlson Comorbidity Index
